# Supplementary material for: A Likelihood Approach for Real-Time Calibration of Stochastic Compartmental Epidemic Models
Source: PLoS Comput Biol. 2017 Jan 17;13(1):e1005257. doi: 10.1371/journal.pcbi.1005257 (PMC5240920; doi:10.1371/journal.pcbi.1005257)
Supplement: S1 File — (TAR.GZ) [file pcbi.1005257.s014.tar.gz › HSPH_Online-SI-Revision/MSS10/n1-mild/n1-mild_NEW2_table-4.pdf]

1	newly 3 weeks spec	24.	32.05	{13., 49.}	55.5417	0.226718	newly 3 weeks cumul	78.	81.21	{41., 115.}	32.3718	0.149052	newly 5 weeks spec	32.	35.27	{11., 56.}	42.6563	0.243533	newly 5 weeks cumul	149.	151.45	{64., 225.}	32.2752	0.162726	newly 59 weeks cumul	331.	497.29	{312., 721.}	60.4683	0.23327
2	newly 3 weeks spec	34.	15.27	{1., 32.}	57.6176	0.618312	newly 3 weeks cumul	90.	61.09	{18., 107.}	41.6778	0.288887	newly 5 weeks spec	17.	8.92	{0., 20.}	59.5294	0.787827	newly 5 weeks cumul	145.	81.71	{25., 144.}	46.3379	0.352514	newly 55 weeks cumul	202.	105.85	{26., 191.}	49.1832	0.403884
3	newly 3 weeks spec	20.	28.44	{0., 61.}	115.	0.846585	newly 3 weeks cumul	57.	120.32	{8., 262.}	153.439	0.482093	newly 5 weeks spec	25.	11.93	{0., 37.}	79.24	1.27143	newly 5 weeks cumul	111.	150.47	{8., 321.}	93.3423	0.470823	newly 64 weeks cumul	238.	171.46	{8., 362.}	53.3613	0.505607
4	newly 3 weeks spec	15.	24.25	{0., 58.}	133.133	0.883751	newly 3 weeks cumul	42.	83.94	{9., 166.}	136.524	0.418879	newly 5 weeks spec	9.	15.69	{0., 44.}	172.111	1.13304	newly 5 weeks cumul	71.	117.52	{9., 235.}	114.817	0.454041	newly 58 weeks cumul	398.	178.21	{9., 416.}	60.3141	0.679233
5	newly 3 weeks spec	50.	18.49	{0., 45.}	69.06	1.03677	newly 3 weeks cumul	122.	68.23	{10., 138.}	54.6311	0.463845	newly 5 weeks spec	37.	9.61	{0., 32.}	78.0811	1.54333	newly 5 weeks cumul	231.	91.05	{10., 194.}	63.1212	0.623057	newly 60 weeks cumul	369.	109.92	{10., 259.}	70.2114	0.76542
6	newly 3 weeks spec	26.	11.03	{0., 22.}	77.8077	0.878772	newly 3 weeks cumul	47.	24.13	{3., 44.}	71.8936	0.587829	newly 5 weeks spec	37.	14.03	{0., 34.}	70.8378	1.07343	newly 5 weeks cumul	121.	50.85	{4., 105.}	71.8264	0.704053	newly 64 weeks cumul	400.	390.95	{4., 787.}	73.3825	0.732745
7	newly 3 weeks spec	25.	58.71	{26., 88.}	145.56	0.429846	newly 3 weeks cumul	44.	271.52	{123., 468.}	521.818	0.756608	newly 5 weeks spec	48.	34.56	{0., 78.}	66.3333	0.95207	newly 5 weeks cumul	142.	348.07	{219., 485.}	150.725	0.413812	newly 63 weeks cumul	381.	512.51	{312., 726.}	47.2257	0.203469
8	newly 3 weeks spec	13.	14.	{5., 25.}	47.8462	0.247127	newly 3 weeks cumul	44.	37.39	{16., 52.}	31.6591	0.177764	newly 5 weeks spec	14.	16.49	{5., 27.}	56.3571	0.24833	newly 5 weeks cumul	78.	69.73	{29., 99.}	34.5513	0.188708	newly 55 weeks cumul	449.	391.68	{147., 531.}	24.3207	0.17782
9	newly 3 weeks spec	32.	24.72	{0., 51.}	57.	0.617677	newly 3 weeks cumul	72.	79.48	{15., 154.}	60.3333	0.336082	newly 5 weeks spec	42.	16.32	{0., 37.}	64.619	1.01737	newly 5 weeks cumul	158.	116.95	{15., 233.}	47.6139	0.399715	newly 54 weeks cumul	322.	157.58	{15., 306.}	52.4224	0.54292
10	newly 3 weeks spec	37.	36.47	{18., 52.}	25.8649	0.163818	newly 3 weeks cumul	113.	110.03	{64., 141.}	20.6991	0.116018	newly 5 weeks spec	27.	32.96	{16., 49.}	43.5556	0.219281	newly 5 weeks cumul	184.	177.59	{103., 228.}	20.8424	0.120508	newly 55 weeks cumul	266.	384.83	{184., 541.}	58.282	0.2296
11	newly 3 weeks spec	20.	6.88	{0., 16.}	69.	0.768051	newly 3 weeks cumul	44.	20.07	{7., 38.}	59.2045	0.473208	newly 5 weeks spec	18.	6.7	{0., 17.}	68.2222	0.854568	newly 5 weeks cumul	86.	34.	{8., 72.}	63.7674	0.556757	newly 54 weeks cumul	455.	124.11	{8., 301.}	73.3912	0.840865
12	newly 3 weeks spec	14.	5.87	{1., 11.}	61.3571	0.583205	newly 3 weeks cumul	27.	16.6	{7., 25.}	42.2963	0.282506	newly 5 weeks spec	18.	6.35	{0., 13.}	66.8333	0.725144	newly 5 weeks cumul	68.	28.44	{11., 44.}	59.8529	0.455595	newly 55 weeks cumul	321.	152.21	{18., 300.}	53.3427	0.538059
13	newly 3 weeks spec	16.	18.81	{0., 35.}	71.9375	0.503347	newly 3 weeks cumul	60.	70.4	{18., 122.}	54.0333	0.262126	newly 5 weeks spec	15.	9.21	{0., 22.}	67.2667	0.856637	newly 5 weeks cumul	111.	91.64	{18., 166.}	42.2883	0.288445	newly 56 weeks cumul	193.	123.95	{19., 216.}	51.9223	0.400434
14	newly 3 weeks spec	29.	43.54	{1., 71.}	90.069	0.499147	newly 3 weeks cumul	93.	128.28	{25., 190.}	63.6774	0.26958	newly 5 weeks spec	30.	37.34	{0., 72.}	86.2	0.723432	newly 5 weeks cumul	159.	207.06	{45., 326.}	65.195	0.305351	newly 60 weeks cumul	260.	385.62	{47., 726.}	91.2692	0.391555
15	newly 3 weeks spec	25.	31.74	{0., 71.}	82.8	0.553785	newly 3 weeks cumul	71.	102.19	{20., 185.}	76.662	0.301962	newly 5 weeks spec	20.	20.14	{0., 44.}	74.5	0.736407	newly 5 weeks cumul	129.	147.87	{20., 266.}	58.5504	0.30648	newly 60 weeks cumul	389.	216.21	{20., 370.}	50.4447	0.455422
16	newly 3 weeks spec	23.	44.71	{25., 62.}	98.7391	0.288901	newly 3 weeks cumul	52.	117.23	{77., 148.}	126.327	0.342144	newly 5 weeks spec	7.	49.22	{27., 67.}	603.143	0.820425	newly 5 weeks cumul	83.	213.38	{133., 271.}	158.024	0.398322	newly 58 weeks cumul	349.	585.25	{414., 704.}	68.6046	0.217719
17	newly 3 weeks spec	17.	22.54	{7., 40.}	70.8235	0.293383	newly 3 weeks cumul	43.	54.13	{19., 87.}	55.0465	0.212205	newly 5 weeks spec	15.	30.15	{3., 56.}	129.933	0.449095	newly 5 weeks cumul	78.	111.88	{32., 197.}	69.2821	0.24792	newly 61 weeks cumul	332.	527.57	{80., 759.}	82.7018	0.331453
18	newly 3 weeks spec	18.	15.8	{8., 22.}	27.5556	0.146148	newly 3 weeks cumul	52.	45.99	{32., 61.}	18.8654	0.0999161	newly 5 weeks spec	18.	16.06	{8., 23.}	27.5556	0.145594	newly 5 weeks cumul	99.	76.9	{52., 99.}	23.7576	0.13128	newly 54 weeks cumul	320.	299.1	{178., 383.}	19.725	0.0990165
19	newly 3 weeks spec	40.	95.66	{73., 116.}	139.7	0.372323	newly 3 weeks cumul	125.	246.53	{207., 291.}	97.928	0.292335	newly 5 weeks spec	35.	85.95	{67., 105.}	149.229	0.406705	newly 5 weeks cumul	206.	427.11	{357., 488.}	108.277	0.315568	newly 61 weeks cumul	389.	746.82	{700., 816.}	94.0308	0.289164
20	newly 3 weeks spec	37.	38.84	{0., 81.}	63.027	0.549807	newly 3 weeks cumul	71.	114.23	{13., 218.}	89.8451	0.353441	newly 5 weeks spec	33.	27.46	{0., 53.}	50.9091	0.647603	newly 5 weeks cumul	150.	175.44	{19., 327.}	58.0533	0.340898	newly 58 weeks cumul	270.	292.67	{27., 512.}	51.0407	0.359637
21	newly 3 weeks spec	20.	7.02	{0., 21.}	76.3	1.28169	newly 3 weeks cumul	49.	35.1	{3., 78.}	58.	0.455936	newly 5 weeks spec	20.	3.18	{0., 15.}	88.2	1.86946	newly 5 weeks cumul	108.	43.01	{3., 98.}	64.3426	0.671268	newly 58 weeks cumul	233.	48.34	{3., 100.}	79.8884	0.975421
22	newly 3 weeks spec	7.	30.88	{14., 51.}	344.857	0.612711	newly 3 weeks cumul	28.	72.16	{36., 111.}	161.214	0.39011	newly 5 weeks spec	13.	42.79	{15., 71.}	235.923	0.504944	newly 5 weeks cumul	64.	153.19	{68., 236.}	144.641	0.365416	newly 60 weeks cumul	439.	658.73	{455., 803.}	53.246	0.191785
23	newly 3 weeks spec	19.	14.38	{0., 40.}	82.6316	0.993116	newly 3 weeks cumul	44.	63.09	{7., 129.}	93.5227	0.397465	newly 5 weeks spec	23.	9.19	{0., 28.}	76.3043	1.42642	newly 5 weeks cumul	99.	83.96	{7., 190.}	62.2626	0.452366	newly 55 weeks cumul	317.	104.64	{7., 251.}	67.1987	0.758549
24	newly 3 weeks spec	25.	23.4	{13., 33.}	28.56	0.143236	newly 3 weeks cumul	65.	60.6	{34., 86.}	23.7231	0.115932	newly 5 weeks spec	28.	28.92	{15., 41.}	30.3571	0.139085	newly 5 weeks cumul	129.	117.14	{72., 159.}	24.4186	0.11976	newly 57 weeks cumul	399.	583.08	{455., 671.}	47.5288	0.165877
25	newly 3 weeks spec	15.	17.53	{7., 28.}	44.2	0.237733	newly 3 weeks cumul	35.	53.19	{31., 71.}	60.3143	0.189769	newly 5 weeks spec	9.	17.98	{1., 32.}	122.889	0.467119	newly 5 weeks cumul	63.	89.54	{48., 131.}	54.6984	0.194012	newly 54 weeks cumul	293.	381.67	{84., 513.}	56.8908	0.27619
26	newly 3 weeks spec	28.	35.88	{23., 50.}	38.2857	0.135833	newly 3 weeks cumul	64.	101.17	{68., 141.}	59.1094	0.188067	newly 5 weeks spec	24.	34.04	{18., 50.}	53.1667	0.187488	newly 5 weeks cumul	119.	170.83	{115., 228.}	46.7311	0.155934	newly 57 weeks cumul	217.	435.95	{308., 614.}	101.535	0.291171
27	newly 3 weeks spec	24.	28.77	{15., 48.}	43.4583	0.165704	newly 3 weeks cumul	69.	73.39	{44., 110.}	32.971	0.136016	newly 5 weeks spec	20.	34.	{17., 51.}	77.6	0.236862	newly 5 weeks cumul	118.	139.82	{80., 213.}	33.9153	0.128278	newly 54 weeks cumul	267.	562.37	{419., 653.}	111.479	0.320091
28	newly 3 weeks spec	11.	10.68	{0., 25.}	72.1818	0.523246	newly 3 weeks cumul	24.	25.03	{4., 56.}	69.625	0.371046	newly 5 weeks spec	16.	17.1	{0., 39.}	80.875	0.57436	newly 5 weeks cumul	66.	55.1	{7., 135.}	63.303	0.431558	newly 60 weeks cumul	343.	509.25	{19., 769.}	81.8863	0.45161
29	newly 3 weeks spec	36.	35.26	{18., 52.}	30.3889	0.204442	newly 3 weeks cumul	105.	99.21	{62., 140.}	26.3524	0.139074	newly 5 weeks spec	22.	36.87	{11., 52.}	85.7727	0.361761	newly 5 weeks cumul	167.	172.43	{107., 241.}	26.8084	0.140274	newly 57 weeks cumul	288.	499.03	{234., 678.}	85.1771	0.30177
30	newly 3 weeks spec	28.	56.6	{32., 80.}	109.357	0.33358	newly 3 weeks cumul	83.	149.8	{96., 196.}	83.6627	0.253324	newly 5 weeks spec	27.	58.2	{27., 83.}	125.852	0.422277	newly 5 weeks cumul	148.	267.28	{157., 342.}	85.4324	0.261814	newly 61 weeks cumul	335.	563.55	{348., 689.}	76.3851	0.255207
31	newly 3 weeks spec	56.	52.97	{37., 71.}	20.6964	0.100216	newly 3 weeks cumul	151.	145.34	{105., 188.}	16.0265	0.0740836	newly 5 weeks spec	34.	53.8	{36., 68.}	61.7647	0.206184	newly 5 weeks cumul	244.	254.87	{190., 327.}	17.2828	0.0749689	newly 59 weeks cumul	353.	598.57	{435., 688.}	70.2918	0.226108
32	newly 3 weeks spec	11.	27.6	{17., 37.}	150.909	0.379247	newly 3 weeks cumul	27.	67.42	{49., 84.}	149.704	0.386421	newly 5 weeks spec	24.	39.18	{24., 54.}	67.3333	0.21437	newly 5 weeks cumul	76.	139.54	{100., 175.}	84.2632	0.255853	newly 57 weeks cumul	391.	727.24	{580., 784.}	85.9949	0.266669
33	newly 3 weeks spec	37.	32.54	{15., 49.}	28.4865	0.177989	newly 3 weeks cumul	84.	89.1	{51., 131.}	26.0476	0.121083	newly 5 weeks spec	32.	38.59	{15., 59.}	47.5313	0.257754	newly 5 weeks cumul	159.	162.85	{94., 232.}	26.4717	0.135739	newly 58 weeks cumul	332.	494.47	{251., 688.}	63.4849	0.248804
34	newly 3 weeks spec	13.	14.05	{0., 35.}	83.1538	0.565899	newly 3 weeks cumul	35.	45.22	{8., 96.}	77.3143	0.310393	newly 5 weeks spec	9.	11.03	{0., 29.}	109.667	0.842323	newly 5 weeks cumul	63.	69.13	{8., 153.}	71.6349	0.359214	newly 57 weeks cumul	230.	104.17	{8., 233.}	58.8913	0.592045
35	newly 3 weeks spec	24.	48.34	{34., 67.}	101.5	0.289833	newly 3 weeks cumul	77.	118.14	{86., 155.}	53.5065	0.176672	newly 5 weeks spec	30.	59.52	{41., 79.}	98.6	0.284501	newly 5 weeks cumul	154.	234.17	{176., 304.}	52.1234	0.173298	newly 59 weeks cumul	405.	710.47	{637., 782.}	76.3235	0.244848
36	newly 3 weeks spec	51.	61.29	{44., 80.}	26.451	0.100465	newly 3 weeks cumul	141.	151.79	{118., 185.}	17.0709	0.0706058	newly 5 weeks spec	46.	73.11	{47., 92.}	60.3696	0.195848	newly 5 weeks cumul	242.	294.45	{229., 349.}	25.5992	0.0975194	newly 61 weeks cumul	339.	737.16	{597., 819.}	117.451	0.334654
37	newly 3 weeks spec	18.	22.53	{0., 56.}	94.2778	0.667889	newly 3 weeks cumul	52.	76.	{13., 154.}	83.5769	0.323594	newly 5 weeks spec	33.	15.27	{0., 39.}	71.9697	1.23023	newly 5 weeks cumul	122.	109.72	{13., 218.}	59.3279	0.383586	newly 57 weeks cumul	268.	150.57	{13., 332.}	57.9739	0.538163
38	newly 3 weeks spec	43.	65.53	{45., 85.}	58.3023	0.238579	newly 3 weeks cumul	95.	170.98	{122., 214.}	83.9368	0.275039	newly 5 weeks spec	60.	66.01	{42., 84.}	25.55	0.156401	newly 5 weeks cumul	220.	304.71	{212., 377.}	44.8773	0.182881	newly 61 weeks cumul	371.	638.62	{492., 775.}	78.0647	0.277866
39	newly 3 weeks spec	35.	32.48	{7., 60.}	47.8286	0.332788	newly 3 weeks cumul	95.	102.37	{38., 188.}	46.7895	0.223021	newly 5 weeks spec	16.	22.2	{1., 41.}	80.375	0.453631	newly 5 weeks cumul	151.	151.27	{43., 259.}	41.4901	0.213084	newly 59 weeks cumul	278.	225.79	{57., 338.}	31.9604	0.214601
40	newly 3 weeks spec	40.	29.1	{0., 68.}	63.8	0.848575	newly 3 weeks cumul	91.	94.2	{7., 203.}	63.8901	0.392885	newly 5 weeks spec	51.	18.79	{0., 49.}	65.8235	1.17282	newly 5 weeks cumul	193.	138.85	{7., 283.}	50.6891	0.460385	newly 62 weeks cumul	329.	178.24	{7., 352.}	49.921	0.548929
41	newly 3 weeks spec	26.	58.46	{30., 87.}	138.231	0.430417	newly 3 weeks cumul	100.	148.14	{87., 191.}	56.7	0.194809	newly 5 weeks spec	38.	68.29	{10., 101.}	104.132	0.467482	newly 5 weeks cumul	177.	283.52	{146., 365.}	71.0734	0.242867	newly 63 weeks cumul	284.	649.16	{187., 837.}	142.479	0.401041
42	newly 3 weeks spec	58.	31.49	{14., 47.}	49.5345	0.36672	newly 3 weeks cumul	126.	75.38	{35., 107.}	45.4127	0.290493	newly 5 weeks spec	44.	40.83	{20., 62.}	30.7955	0.210459	newly 5 weeks cumul	229.	152.21	{78., 231.}	38.1354	0.248621	newly 62 weeks cumul	291.	640.03	{503., 759.}	123.845	0.371158
43	newly 3 weeks spec	61.	55.88	{4., 124.}	62.8197	0.50475	newly 3 weeks cumul	155.	189.83	{51., 335.}	59.6581	0.265446	newly 5 weeks spec	49.	36.21	{0., 89.}	68.3469	0.856972	newly 5 weeks cumul	274.	272.09	{77., 551.}	51.4051	0.284094	newly 64 weeks cumul	392.	356.66	{83., 812.}	57.4592	0.34357
44	newly 3 weeks spec	34.	19.2	{0., 56.}	70.0588	1.02648	newly 3 weeks cumul	64.	67.18	{9., 135.}	65.125	0.356853	newly 5 weeks spec	35.	11.94	{0., 34.}	76.4	1.39264	newly 5 weeks cumul	143.	94.83	{9., 203.}	56.5524	0.469518	newly 56 weeks cumul	211.	115.98	{9., 265.}	57.346	0.547826
45	newly 3 weeks spec	33.	22.45	{3., 43.}	49.2424	0.380878	newly 3 weeks cumul	81.	70.97	{23., 124.}	41.1235	0.232191	newly 5 weeks spec	24.	14.88	{0., 31.}	56.5	0.590328	newly 5 weeks cumul	141.	104.47	{29., 192.}	41.4965	0.277073	newly 54 weeks cumul	301.	163.43	{31., 276.}	49.5316	0.39249
46	newly 3 weeks spec	19.	43.72	{22., 73.}	134.316	0.336709	newly 3 weeks cumul	57.	99.06	{53., 154.}	79.6491	0.234115	newly 5 weeks spec	23.	57.64	{33., 85.}	152.609	0.379018	newly 5 weeks cumul	104.	208.8	{112., 326.}	103.942	0.287496	newly 61 weeks cumul	451.	722.11	{620., 861.}	60.5743	0.202686
47	newly 3 weeks spec	5.	16.04	{6., 26.}	225.2	0.457306	newly 3 weeks cumul	19.	38.08	{18., 61.}	105.158	0.283234	newly 5 weeks spec	12.	24.16	{9., 39.}	114.333	0.312901	newly 5 weeks cumul	47.	82.26	{38., 139.}	84.1702	0.243412	newly 59 weeks cumul	335.	623.84	{450., 754.}	86.806	0.263147
48	newly 3 weeks spec	15.	23.39	{11., 34.}	69.1333	0.273172	newly 3 weeks cumul	52.	66.9	{41., 89.}	40.6538	0.164842	newly 5 weeks spec	38.	22.92	{7., 34.}	40.2632	0.350415	newly 5 weeks cumul	137.	112.47	{73., 142.}	20.7518	0.133757	newly 54 weeks cumul	271.	405.93	{102., 528.}	72.8672	0.307953
49	newly 3 weeks spec	12.	12.88	{3., 24.}	63.	0.362732	newly 3 weeks cumul	36.	33.49	{12., 59.}	43.75	0.232984	newly 5 weeks spec	11.	15.6	{2., 31.}	83.4545	0.395472	newly 5 weeks cumul	67.	62.81	{20., 104.}	44.3134	0.243847	newly 58 weeks cumul	292.	348.72	{31., 534.}	61.1507	0.357103
50	newly 3 weeks spec	24.	35.54	{25., 45.}	50.1667	0.169699	newly 3 weeks cumul	70.	97.36	{81., 117.}	40.2	0.143453	newly 5 weeks spec	19.	36.52	{26., 47.}	93.6842	0.276419	newly 5 weeks cumul	120.	169.99	{139., 199.}	42.9417	0.152523	newly 55 weeks cumul	321.	511.96	{312., 599.}	61.9751	0.203441
